# “Tudo começa no acolhimento”: acesso, aceitabilidade e elementos para a promoção da saúde de adolescentes e jovens na atenção primária

**DOI:** 10.1590/0102-311XPT007526

**Published:** 2026-08-31

**Authors:** Jamile Guimarães, Valéria Monteiro Mendes, Isabella Silva de Almeida, José Ricardo de Carvalho Mesquita Ayres

**Affiliations:** 1 Universidade Federal de Minas Gerais, Belo Horizonte, Brasil.; 2 Universidade de São Paulo, São Paulo, Brasil.

**Keywords:** Atenção Primária à Saúde, Promoção da Saúde, Direitos Humanos, Primary Health Care, Health Promotion, Human Rights, Atención Primaria de Salud, Promoción de la Salud, Derechos Humanos

## Abstract

O artigo apresenta um estudo qualitativo sobre o acesso de jovens a serviços públicos de saúde nas cidades de Santos, São Paulo e Sorocaba, Estado de São Paulo, Brasil. O objetivo foi identificar a significação atribuída às experiências de acesso para caracterizar um conjunto de critérios de aceitabilidade de práticas de atenção à saúde. Os instrumentos metodológicos utilizados foram a observação do cotidiano em unidades básicas de saúde e nos territórios adscritos, e 34 entrevistas semiestruturadas com jovens, entre 15 e 24 anos. Além de barreiras estruturais que restringem a presença nesses serviços (elevado tempo de espera, dificuldade em agendamento de consulta e desfechos insatisfatórios na atenção), as narrativas dos jovens enfatizam o caráter interativo do cuidado, a observância às especificidades da adolescência e o papel dos serviços de saúde na construção de si, mediante o engendramento intersubjetivo de recursos para uma vida mais feliz. Eles afirmam a centralidade de seu reconhecimento como sujeitos de direito, coprodutores de um cuidado integral em saúde, que contribua com aprendizados para o viver. Para tanto, o atendimento satisfatório deve promover a sua participação no processo terapêutico, dispor de informações e orientações que propiciem decisões dialogadas e, numa dimensão ampliada, o desenvolvimento da autonomia.

## Abstract (en)

This article shows a qualitative study on the access of young people to public health services in the cities of Santos, São Paulo, and Sorocaba, São Paulo State, Brazil. The objective was to identify the meaning attributed to access experiences in order to characterize a set of criteria for the acceptability of health care practices. The methodological instruments used were the observation of daily life in the basic health units and in the assigned territories and 34 semi-structured interviews with young people between 15 and 24 years old. In addition to structural barriers that restrict presence in these services (long waiting times, difficulty in scheduling appointments and unsatisfactory care outcomes), the young people’s narratives emphasize the interactive nature of care, the observance of the specificities of adolescence and the role of health services in the construction of themselves, through the intersubjective engendering of resources for a happier life. They affirm the centrality of their recognition as subjects of rights, co-producers of comprehensive health care, which contributes with learning for living. To this end, satisfactory care must promote their participation in the therapeutic process, provide information and guidance that enable dialogued decisions and, in a broader dimension, the development of autonomy.

## Abstract (es)

Se trata de un estudio cualitativo sobre el acceso de los jóvenes a los servicios públicos de salud en las siguientes ciudades: Santos, São Paulo y Sorocaba, Estado de São Paulo, Brasil. Tuvo como objetivo identificar el significado atribuido a las experiencias de acceso para caracterizar un conjunto de criterios de aceptabilidad de las prácticas de atención a la salud. Se utilizaron los siguientes instrumentos metodológicos: observación de la vida cotidiana en las unidades básicas de salud y en los territorios adscritos; y 34 entrevistas semiestructuradas con jóvenes de entre 15 y 24 años. Además de las barreras estructurales que limitan el acceso a estos servicios (elevado tiempo de espera, dificultad para programar citas y resultados insatisfactorios en la atención), las narrativas de los jóvenes resaltan el carácter interactivo de la atención, el respeto por las particularidades de la adolescencia y el papel de los servicios de salud en la construcción de sí mismo, a través del engendramiento intersubjetivo de recursos para una vida más feliz. Ellos afirman la centralidad de su reconocimiento como sujetos de derecho, coproductores de una atención integral en salud, que contribuya a aprendizajes para la vida. Para ello, una atención satisfactoria debe promover su participación en el proceso terapéutico, brindar información y orientación que propicien decisiones dialogadas y, en una dimensión más amplia, el desarrollo de la autonomía.

## Introdução

O Programa Saúde do Adolescente (1989), o Estatuto da Criança e do Adolescente (1990), as Diretrizes Nacionais para a Atenção Integral à Saúde de Adolescentes e Jovens na Promoção, Proteção e Recuperação da Saúde (2010) e o Estatuto da Juventude (2013) representam marcos para a garantia de direitos desses segmentos populacionais. Tais documentos legais/organizativos são responsáveis pela institucionalização do paradigma da proteção integral, que reconhece adolescentes e jovens como sujeitos de direitos.

Esse paradigma é fundamentado pelo princípio da integralidade e por processos de participação social e política, inclusive na formulação, implementação e avaliação de políticas públicas. Conforme esse ideário, a participação articula-se com as concepções de autonomia, cuidado e cidadania, favorecendo a aprendizagem de valores e competências como categorias impulsionadoras para trilharem caminhos emancipatórios. O processo participativo tem como âmago a dinâmica dialógica (intersubjetiva) que se consubstancia em novos sentidos, símbolos e significados pessoais e coletivos ^1^; uma socialização conscientizadora (reflexiva e cidadã), implicada à promoção da saúde, cujo horizonte é a integralidade do cuidado e a melhoria da qualidade de vida.

Todavia, as experiências de políticas públicas para jovens emergentes entre as décadas de 2000 e 2010 foram incipientes; resultaram em projetos isolados que não se consolidaram como formas democráticas de gestão ^2^. Mesmo com avanços oriundos da expansão da cobertura da atenção primária à saúde (APS), a atenção integral à saúde de jovens defronta-se com importantes barreiras ^3^ ^,^ ^4^. Na assistência, predominam as práticas profissionais verticais, baseadas no saber técnico e na concepção de juventude como fase “de risco”, associada a problemas sociais ^5^. Compreender as necessidades de saúde de jovens requer um modelo de atenção ampliado, que incorpore modos de cuidar fundamentados em valores, condutas e conhecimentos culturais como elementos constitutivos das práticas de saúde ^6^.

As ações em saúde voltadas aos mais jovens costumeiramente estão limitadas à contracepção, prevenção às infecções sexualmente transmissíveis (IST)/HIV, gravidez na adolescência e violência intrafamiliar ^7^ ^,^ ^8^. No entanto, cabe ressaltar a potência da APS, especialmente das unidades básicas de saúde (UBS), como espaços de cuidado longitudinal capazes de produzir relações dialógicas e promover redes de atenção integral ^9^.

Mesmo com a revisão da Política Nacional de Promoção da Saúde em 2018, ainda é restrita a discussão sobre a composição de processos avaliativos que enfatizem o significado formativo das práticas de saúde, além de suas finalidades técnicas ^10^. Neste artigo, analisamos as experiências de acesso a serviços de saúde de jovens, a fim de caracterizar os critérios de aceitabilidade de práticas assistenciais. A aceitabilidade decorre de uma abordagem de saúde baseada em direitos humanos ^11^: é constituída na garantia de direitos relativos à identidade da pessoa, às especificidades culturais, à informação e educação, e da participação em decisões sobre a própria vida e saúde. A discussão empreendida traz significados e repercussões no acesso e avança, ao delinear elementos para a produção de um modelo de cuidado alinhado às perspectivas subjetivas, às necessidades e potencialidades de modos de jovens construírem seu viver.

## Método

Trata-se de um estudo qualitativo sobre o acesso de jovens a serviços de saúde em três cidades do Estado de São Paulo, Brasil: Santos, São Paulo e Sorocaba. Esse recorte temático está vinculado a um projeto de intervenção sobre prevenção de IST/HIV, COVID-19 e sofrimentos psicossociais realizado em escolas públicas de ensino. A formação de agentes-jovens visava à educação entre pares, que ensejaria iniciativas de prevenção articuladas nas UBS de referência ^8^. O modelo de “prevenção integral” desenvolvido foi divulgado em seminários e em formações voltados para jovens participantes do projeto, gestores e profissionais da saúde e da educação.

O trabalho de campo foi realizado em 2023, em quatro UBS de São Paulo, uma em Santos e uma em Sorocaba. Os territórios circunscritos às unidades básicas eram periféricos, de padrões socioeconômicos que variavam de situações de alta vulnerabilidade (favelas) e bairros de classe média baixa e média. Os instrumentos metodológicos mobilizados foram a observação do cotidiano do trabalho, em consultas, visitas domiciliares, reuniões de equipe e de grupos, e entrevistas semiestruturadas com gestores, profissionais de saúde e jovens usuários.

As andanças nos territórios permitiram conhecer outros serviços e compreender sobre como jovens se relacionam, socializam e produzem vida e saúde. Entre os serviços visitados, citamos: (a) na saúde: o Centro de Atenção Psicossocial Infantojuvenil (CAPS IJ) e a Casa do Adolescente; (b) na educação: Centro Educacional Unificado (CEU) e escolas de Ensino Médio ligadas ao campo da pesquisa; (c) Na assistência social: Centro para Crianças e Adolescentes (CCA) e Centro da Juventude (CJ), Projeto Jovem Aprendiz e Serviço de Acolhimento Institucional para Crianças e Adolescentes (SAICA); (d) equipamentos do território: organizações não-governamentais (ONGs) e projetos comunitários voltados a adolescentes e jovens.

O número de entrevistados não foi pré-determinado. O critério de elegibilidade primário foi o uso de algum serviço público de saúde local. Também definimos a faixa etária de 15 a 24 anos e buscamos abarcar uma composição diversa de marcadores sociais da diferença. Inicialmente, contamos com o apoio de profissionais das UBS para mediar o contato com os usuários. Face o baixo quantitativo, incluímos jovens das escolas participantes e de projetos sociais e ONGs, identificados em nossas visitas exploratórias.

Neste artigo, analisamos as 34 entrevistas feitas com jovens, entre 15 e 24 anos. A maioria, advinda de famílias dos segmentos populares, se identificou como cisgênero e heterossexual, com algumas identidades dissidentes (1 mulher trans e 1 pessoa não-binária, 4 homossexuais, 3 bissexuais e 1 pansexual). Sendo 19 negros, 14 brancos e 1 indígena.

As entrevistas e notas de campo constituem o material empírico do estudo. Tal *corpus* foi objeto de um tratamento hermenêutico, que gerou a identificação de temas predominantes emergentes, os quais foram consolidados em temas principais, organizados em subcategorias, e casos que definiam suas conceituações e elementos característicos. Foram delimitados os seguintes tópicos: experiências de acesso em serviços públicos de saúde (subdivididas em avaliações positiva e negativa do atendimento); barreiras e potencialidades para o acesso juvenil; aspectos e elementos constituintes do modo de cuidado almejado; especificidades da adolescência e atendimento especializado; necessidades em saúde; apreciação geral sobre os serviços de saúde acessados. Por fim, esses tópicos foram repensados à luz de princípios orientadores de avaliação formativa de projetos de saúde, concebidos por Ayres ^10^: interação, identidade/alteridade, plasticidade, projeto, desejo, temporalidade, não-causalidade e responsabilidade.

Apresentamos o estudo nos serviços e projetos observados. A pesquisa obteve permissão para dispensa parental de consentimento do Comitê de Ética em Pesquisa da instituição coordenadora, o Instituto de Psicologia da Universidade de São Paulo (CAAE 00530918.9.0000.5561): os participantes menores de 18 anos assinaram o Termo de Assentimento Livre e Esclarecido (TALE) e os jovens entre 18 e 24 assinaram o Termo de Consentimento Livre e Esclarecido (TCLE). Nomes fictícios são usados no texto para assegurar o sigilo das conversas e o anonimato dos/as participantes.

## Resultados e discussão

### Experiências nos serviços públicos de saúde: valores e práticas de reconhecimento, acolhimento e vínculo

Com base na observação e nos relatos das/os jovens, mapeamos os serviços públicos e as ofertas mais acessados por elas/es na Rede de Atenção à Saúde. Grande parte da demanda espontânea da UBS é generificada e está relacionada à saúde sexual e reprodutiva: testes de IST/HIV e de gravidez, grupo de planejamento familiar, vacinação contra HPV, além de consultas de pré-natal. Vários/as entrevistados/as acessavam a UBS para acompanhamento ambulatorial em consultas (clínica médica, de enfermagem, odontológica, nutricional, psicológica) e coleta de exames laboratoriais. Aqueles/as que dispunham de plano privado utilizaram esse serviço para vacinação contra a COVID-19.

Para demandas de urgência, a Assistência Médica Ambulatorial (AMA), a Unidade de Pronto Atendimento (UPA) e os hospitais são acessados conforme a proximidade da residência e/ou a percepção da gravidade do caso. Um dos campos dispunha da Casa do Adolescente, que oferece atendimento ambulatorial multidisciplinar; sendo salientada a qualidade da assistência nas especialidades (hebiatria, dermatologia, ginecologia, odontologia, nutrição e psicologia). Um quantitativo diminuto era usuário/a do CAPS IJ, com participação em grupos e consultas com psiquiatra e equipe multiprofissional.

Ao narrarem experiências positivas e negativas de acesso, os/as entrevistados/as apontaram critérios de aceitabilidade da assistência para jovens: acolhimento, vinculação, orientação/informação, encaminhamentos, resolutividade e preparo dos/as profissionais para atendê-las/os.

A ênfase em elementos constitutivos do encontro de sujeitos no e pelo ato de cuidar delineia atitudes e práticas a serem incentivadas nos serviços de saúde. A avaliação positiva decorre do entendimento de ter sido bem tratado/a pelas/os trabalhadoras/es (Quadro 1). A “educação” (cordialidade), a “atenção” e a interatividade (prestar informações e dialogar) emergem como critérios que diferenciam o “bom” e o “mal” atendimento. São parâmetros consonantes à relevância atribuída ao “acolhimento”, considerado princípio fundante do cuidado ao segmento jovem.

**Quadro 1:** Experiências positivas nos serviços de saúde e a significação atribuída.

EXPERIÊNCIA POSITIVA	SIGNIFICAÇÃO ATRIBUÍDA
“*Minha dermatologista é super fofinha, super educada e ela vê tudo bonitinho e me encaminha, conversa comigo. Eu explico as coisas. A interação mostra que a pessoa está preocupada com você. Foi uma das questões que eu gostei de lá*” (Marina, 17 anos)	Satisfação com a participação ativa no processo de cuidado dialógico Percepção de reconhecimento como sujeito no atendimento Proximidade e vínculo Informação acessível e entendimento do procedimento adotado
“*O daqui* [atendimento psicológico da UBS] *foi muito bom porque eles me deram muita atenção, me ajudaram muito* (...) *me trataram bem, quiseram ouvir*” (Alice, 15 anos)	Percepção de si como agente de/no cuidado, com voz ativa no atendimento Sentimento de acolhimento
“*Elas* [profissionais] *disseram que o procedimento* [coleta de Papanicolau] *é muito pesado pra minha idade. Elas me deixaram superconfortável. Fiquei nervosa no começo, mas elas me deixaram bem de boa*” (Joana, 18 anos)	Sentimento de acolhimento Informação sobre o procedimento gera conforto e afirma a sua dignidade no atendimento
“*Na Casa do Adolescente, eles prezam muito por você ser mais independente. A partir de uma certa idade, eles falavam pra você passar nas consultas sozinha* (...)*. Eles tentavam mais falar com o adolescente do que só com a mãe. E acho que dá pra entender até melhor o que a pessoa precisa*” (Liz, 17 anos)	Serviço de saúde como agente de socialização juvenil Participação do serviço de saúde no processo de autonomização dos usuários
“[Os profissionais da Casa do Adolescente] *tratam a gente muito bem, sabe? A gente estando com os pais, com o responsável ou sozinho. Eles perguntam: ‘Você tá bem? Você entendeu? Quer que eu fale de novo?’. É uma paciência, parece que eles até foram estudados pra lidar com adolescentes, pra dar apoio* (...)*. É surreal o apoio que eles dão!*” (Yolanda, 17 anos)	Participação e escuta ativa facultam perceber que é compreendido pelos profissionais nos atendimentos Oferta de orientação/informação como fundamento de produção de conhecimento em saúde e de participação na tomada de decisão sobre o cuidado Suporte social no serviço de saúde
“*Eu sempre frequentei lá, já me conhecem, tem aquela proximidade. Tem aquele certo respeito, intimidade de falar: ‘Poxa, cê tá precisando disso, vou tentar arrumar o mais rápido possível, já vou fazer o encaminhamento’. Acho que quando você tem aquele contato, você já conversa bem*” (Caio, 17 anos)	Estratégia Saúde da Família como qualificadora do acesso e do vínculo Relevância da proximidade entre profissional e usuário para estabelecimento de cuidado longitudinal

O acolhimento é concebido como lastro para a tessitura de um acesso humanista e vinculativo, principalmente, nessa fase da vida. Como ferramenta tecnológica de intervenção parte (e depende) de uma visão humanista dos cuidados de saúde e do adoecimento pelos profissionais de saúde, mediante o referencial da determinação social da saúde e de um posicionamento ético alicerçado nos valores de promoção da saúde ^12^.

Fica patente a expectativa de que a construção do cuidado seja fundamentada no “respeito” demonstrado pelos/as profissionais (adultos). Sobressai a importância conferida à promoção/valorização de sua voz e agenciamento durante o atendimento. A consulta é compreendida como espaço intersubjetivo, no qual o/a jovem é posicionado como sujeito e em que se vislumbra o seu estado de saúde na vida-em-percurso.

Para tanto, é necessário que sejam fornecidas informações sobre a sua questão de saúde e o procedimento/estratégia a ser adotado. Além de ensejar maior segurança em relação à terapêutica, o conhecimento potencializa expressar o que sentem, compreender as orientações e esclarecer dúvidas.

“*Ela* [a hebiatra] *super interage comigo* (...)*. Por exemplo, ela vai lendo meus exames: ‘Essa vitamina tá não sei o quê. Acho que vai ter que tomar de novo, tudo bem?’ - ‘Tudo bem’. Ela vai explicando. Ela conversa comigo. Eu gosto* (...)*. Tem um relatório lá também que eles veem. Aí ela fala: ‘Ah, você ainda tá cursando isso? Você terminou? Como foi?’. ‘Como você tá se sentindo?’, se você tá com algum novo problema que quer pedir encaminhamento. Fala assim. Elas perguntam.* (...) *a nutricionista também. A gente conversou muito sobre as comidas porque a minha mãe é cozinheira* (...)*. Aí eu comecei a explicar pra ela as coisas* (...) *que eu me alimento muito bem, que às vezes não é questão da alimentação, mas sim do psicológico, que acaba me afetando. Acho que a interação, que mostra que a pessoa tá preocupada com você foi uma das questões que eu gostei de lá*” (Marina, 17 anos).

A fala de Marina nos coloca numa cena de atendimento marcada por uma abordagem integral, que possibilita aos profissionais complexificar hipóteses diagnósticas e demandas de tratamentos, ao conectá-las com sua situação de vida. Dessa narrativa emblemática, que igualmente depreendemos como o “respeito” - tantas vezes mencionado pelos/as jovens -, remete ao engajamento em experiências que motivam expressões da autorrealização prática, e afirmam seu reconhecimento social como sentimento do próprio valor, de autoestima e de autoconfiança ^13^. Como assevera Honneth ^13^, os sujeitos humanos precisam de uma estima social que lhes permita referir-se positivamente a suas características e capacidades concretas. A ideia fundamental é que os indivíduos se formam e constituem suas identidades quando são reconhecidos intersubjetivamente. Para isso, é necessário construir modos de operar as práticas de saúde que realizem um autêntico encontro entre sujeitos, no qual inalienáveis interesses de compreensão e simultânea construção do si mesmo e do outro estejam em cena ^10^ ^,^ ^13^.

Atuar nessa perspectiva permite a equipes e à gestão de serviços da APS experimentarem práticas que fomentem o convívio com a alteridade, favorecendo o cuidado mediado por tecnologias relacionais; sem prescindir das contribuições dos saberes técnicos e dos procedimentos/protocolos preconizados por evidências científicas. Trata-se de um delicado movimento de construção/adensamento de vínculos com ressonância em erigir certa autonomia no cuidado de si pelas/os jovens ^10^ ^,^ ^14^.

As narrativas de experiências negativas de atendimento (Quadro 2) igualmente sinalizam ações esperadas/buscadas/desejadas nos encontros entre os/as profissionais e os/as jovens. A ausência de conexão com os/as profissionais da saúde é destacada como barreira à construção do acolhimento e do vínculo. Um exemplo relativamente comum foi o do profissional focado na tela do computador durante a consulta, assimilado como não-centrado no usuário e na sua situação de saúde/doença.

**Quadro 2:** Experiências negativas nos serviços de saúde e a significação/efeitos.

EXPERIÊNCIA NEGATIVA	SIGNIFICAÇÃO/EFEITOS
“*Com o médico do Implanon eu fiquei com vergonha. Eu tenho que esperar pra colocar o Implanon. Ele não deu opção. Eu fico com medo de tomar* [injeção] *de 3 em 3 meses. Mas eu fiquei com vergonha* [de falar] *porque tinha muita gente na sala. Estava eu e meu namorado, eles* [os profissionais] *fizeram um monte de perguntas. Eu não sou de falar sobre esse assunto. Tinha uns quatro, cinco homens* [participando da atividade]*. Eu também não entendi por que eu estava na sala com um monte de gente. Não explicaram nada. Eu queria perguntar, mas era um monte de gente na sala.* (...) *se eu vier de 3 em 3 meses, eu vou acabar esquecendo. E eu esqueci, né? E eu tô evitando fazer aquelas ‘coisa’* [relação sexual]*. Aí, eu preferi guardar. Quando eu saí do posto, eu pesquisei e entendi*” (Andreia, 17 anos)	Ausência de informação sobre uma situação não usual e sobre o método contraceptivo inicialmente adotado gera desconforto e constrangimento e, por conseguinte, inibição na comunicação Falta de consentimento sobre a observação da consulta por terceiros obstaculiza a cumplicidade no encontro profissional-usuário Falta de orientação pelo profissional ocasiona a busca de informações na internet Sem escuta, a usuária não pode expressar o desejo/expectativa que a levou ao serviço nem expor incompatibilidade entre o método de contracepção recebido e suas práticas e atitudes
“*Eu entrei na sala* [dentista]*, minha amiga estava conversando comigo, que eu estava com medo. Ela tava tentando me acalmar. Ela* [dentista] *falou que se minha amiga não parasse de falar, ia tirar ela da sala. Depois daquilo, não quis mais vir aqui. Vou pra outro dentista, quando eu tô com dor de dente*” (Andreia, 17 anos)	Desistência de utilização da especialidade no serviço pela conduta da profissional
“*Eu tava com medo. Eu vi um vídeo no YouTube, eu vim aqui na UBS* [para entender como seria]*. Foi a enfermeira que colheu o meu Papanicolau. Na hora doeu muito! Muito, muito. Eu senti muita dor, mas eu aguentei. Ela só me orientou como seria e falou que meu útero tava tudo ok. Eu acho que poderia ter sido diferente se ela perguntasse ‘Você está sentindo alguma dor?’. Eu falaria* (...) *Ou falar: ‘Talvez doa um pouco, porque você nunca fez’. Acho que se eu fosse uma pessoa, vamos supor, mais rica, talvez o médico ou a enfermeira orientaria melhor do que uma pessoa assim, pobre*” (Juliana, 16 anos)	Entendimento de que a qualidade do atendimento (manejo mais cuidadoso e prestação de orientações consistentes e completas) varia conforme a classe social, gerando desconfiança em relação ao sistema de saúde
“*Se eu for no SUS, eles vão me dar remédio e é isso. Às vezes não é isso que eu preciso, às vezes eu preciso de um encaminhamento pra fazer um exame de endoscopia, e aí vai demorar não sei quanto tempo. Até lá, já mudou - já posso tá pior, já posso tá melhor*” (Marina, 17 anos) “*Não vou porque eu sei que eles vão só me medicar, não vão procurar saber a fundo, e se for pra medicar, eu consigo me medicar em casa*” (Alessandra, 17 anos)	Adoção do modelo queixa-conduta em serviços de urgência/emergência é considerada insuficiente, podendo ocasionar a busca por consultas em serviços privados de atendimento rápido e com custo baixo, mas de pouca confiabilidade quanto ao tratamento em médio prazo Ausência de diagnóstico somada à exclusiva prescrição de medicamentos pode resultar na opção por automedicação

SUS: Sistema Único de Saúde; UBS: unidade básica de sáude.

A maioria das/os jovens expressou desejo/pretensão de receber um atendimento humanista. Variantes da frase “eles nem olham na minha cara direito” sintetizam o “desconforto” e o descontentamento ocasionados pela impessoalidade, entendida como indiferença. É emblemático o trecho da entrevista com Hugo, 19 anos, em que narra sua experiência em um AMA: “*Desde o atendimento* [na recepção]*, não fiquei confortável porque não teve isso de olhar na cara, de perguntar o que eu tava sentindo. Não teve esse acolhimento. Quando eu fui atendido também. Ele* [médico] *só perguntou meu nome. Falou pra mim: ‘Senta aí’. Aí colocou o negócio* [estetoscópio] *no meu peito e* (...)*. Isso sem olhar pra minha cara. Anotou* [receita] *e falou ‘Você tá liberado. Eu vou passar os antibióticos’*”.

As críticas ao modo como ocorrem os atendimentos nos serviços de saúde destacam a necessidade de uma postura empática como ponto de partida para a construção do vínculo. A “empatia” diz respeito à compreensão do outro pela/o profissional. Uma compreensão integral do sujeito em sua situação de vida - muitas vezes marcada por conflitos e sofrimentos na família e/ou na escola -, sendo princípio/valor essencial do cuidado em saúde. Nas palavras de Júlia, 19 anos: “*Tem que ter esse olhar, né? Tipo, você não sabe o que tá passando no outro*”.

Mais do que cordialidade, as expressões corriqueiras “*Como você tá?*”, “*O que você tá sentindo?*”, foram apontadas como imprescindíveis e potentes para se acessar sentimentos, emoções, anseios e entraves ao bem-estar. Igualmente, são elementos-meio para demonstrar disponibilidade à escuta, pontuada de forma recorrente como aspecto fundamental ao trabalho nos serviços; não apenas para compreender a realidade vivida com base em saberes e experiências da/na comunidade, mas sobretudo para conceber ações de cuidado alinhados (dinamicamente) com as necessidades, interesses e possibilidades de cada indivíduo, sua família e/ou território.

Eles/as enfaticamente rejeitavam sentir que são “só mais um, só mais um paciente que tá lá”, também expressa na descortesia e na “falta de paciência”, que produz “desconforto” no atendimento. Ariel, 17 anos, assinala a relevância da receptividade e do acolhimento para que os/as jovens se sintam confortáveis em “*procurar o médico*” e minorar “*barreiras mentais*” que impedem “*a pessoa de ir* [pelo pensamento]*, ah, eu não tô tão mal assim!*”. Embora saliente que o “*cansaço*” e a “*apatia*” das/os profissionais de saúde devam ser reconhecidos como consequentes do processo de trabalho precarizado, Ariel finaliza sua reflexão reforçando que: “*Eu só vou no médico quando eu estiver realmente muito ruim*”.

### Especificidades da adolescência e práticas de cuidado: perspectivas para a qualificação dos profissionais da saúde

Diante da questão “Você acha que os serviços de saúde estão preparados para cuidar da saúde de adolescentes e jovens?”, a maioria das/os entrevistadas/os considerou haver falta de preparo dos/as profissionais para a atenção, assinalando a necessidade de qualificação especializada.

Para que o encontro terapêutico seja efetivo é fundamental que sejam compreendidas as especificidades dessa fase. Alessandra, 17 anos, afirma que: “*enquanto fase marcada por intensos processos de mudanças, a adolescência traz desafios adicionais a uma saúde pública precarizada*”. Em consonância, Júlia, 19 anos, evoca a necessidade de os/as profissionais terem “*formação para lidar com adolescentes*”, a fim de fornecer “*apoio*” frente às dificuldades que enfrentam. Eles/as consideram adolescentes “*complicados*”, assinalando algumas características: são “*muito sentimentais*”, “*muito cabeça dura*”, além de “*situações psicológicas*” (como “*medo*”, “*vergonha*”, “*pensam demais*”) e a experiência de estar “*se descobrindo, se conhecendo*”.

O diálogo compreensivo e construtivo é considerado prática basilar na assistência à saúde, e deve ser incorporado como dimensão essencial à avaliação em saúde. Yolanda, 17 anos, argumenta em favor da “*conversa*” para “*entender mais, se aprofundar* [com] *essa pessoa*”, já que “*cada adolescente tem a sua forma de ser*”. Essa jovem se ressente da ausência de “*diálogo*” nas consultas nos vários serviços públicos que acessou. Ela asseverou que a deferência/zelo aos sentimentos, ideias e situação do/a jovem usuário/a possibilitaria ao profissional organizar o modo de atenção e adequá-lo ao momento presente, tal como exemplificou: “*Igual o dia que o CAPS foi em casa. Eu tinha acabado de ter uma crise, então, tava me sentindo mal, tava chorando, e naquele dia eu não queria falar, queria o meu tempo. E ela meio que insistiu. Foi no meu quarto e aí eu fiquei assim: ‘Vou ter que falar’ - mas obrigadamente*”.

Depreende-se, assim, a atenção à plasticidade do cuidado: é preciso saber/aprender a adaptar/transformar discursos e práticas às histórias de vida dos/as usuários, circunstâncias e expressões do presente ^10^. A plasticidade é fundamental para dirimir dificuldades inerentes aos encontros do cuidar entre alteros. De modo geral, o processo de cuidado ainda padece da estratificação colocada pelo paradigma biomédico, que concebe o sujeito como alguém acometido por uma doença ^14^. A reivindicação é que o cuidado a ser produzido (com elas/es) reconheça tanto a singularidade do eu quanto as especificidades da adolescência, sem estabelecer um padrão que vele a diversidade de biografias, trajetórias, concepções e possibilidades.

“*Às vezes colocar uma padronização no adolescente não é uma coisa muito boa porque você vai abordar o adolescente com o mesmo pensamento que você abordou o adolescente anterior. Então é mais entender que cada adolescente tem a sua forma de se expressar, sua forma de viver*” (Josiane, 15 anos).

Outras considerações relevantes sobre a assistência voltada a adolescentes e jovens, foram: (1) o direito ao atendimento desacompanhado, a fim de garantir sua privacidade e liberdade de expressão e (2) o provimento de recursos humanos “suficientes” para atuar em suas necessidades de saúde. Eles referem especialmente ao acompanhamento psicológico, reiteradamente mencionado como a sua principal necessidade em saúde.

A identificação do atendimento psicológico como “apoio” sintetiza a centralidade da demanda por suporte social a determinadas questões existenciais e sofrimentos; remete a uma vacância de coesão e solidariedade nas redes sociais e comunitárias. O crescente individualismo na sociedade neoliberal tem gerado uma “solidão não desejada” entre pessoas jovens ^15^. Além da crescente hiperconexão e da digitalização nas relações entre pares, a precarização da vida afeta decisivamente as relações familiares, intensificando os desafios de produzir a si próprios como sujeitos capazes de viver autonomamente, numa fase da vida já marcada por incertezas e hesitações ^16^: “...*nem todos os jovens sabem o que tem que fazer na vida, então eu acho que deveriam receber mais conselho, mais ajuda, mais atenção*...” (Ana, 17 anos).

Essas falas localizam o significado atribuído ao “psicólogo”: um profissional referido, por quase todos/as, como alguém que “*conversa*”, “*entende*”, “*orienta*” e oferece um espaço de “*liberdade para falar o quiser*”. Convergem com a perspectiva formativa, que defende que as identidades dos sujeitos são também construídas em processos de interação intersubjetiva nos atendimentos em serviços de saúde ^10^. A leitura que fazem do “apoio do psicólogo” não se restringe a uma escuta acolhedora, serve, sobretudo, à produção conjunta de recursos para lidarem com seus problemas e promotores de bem-estar. A ideia é que a “terapia” permitiria a desconstrução de ideias e práticas, levando-os/as a “*se olhar com um olho diferente. A gente pensa ser uma pessoa mais errônea do que a gente é realmente*” (Hugo, 17 anos). Apenas sublinhamos o quantitativo baixo de participantes do estudo que tiveram algum acompanhamento psicológico (4 de 34 aludem certa idealização).

As narrativas asseveram a observância do desejo como princípio orientador das práticas de saúde. O desejo refere-se às pretensões e perspectivas de futuro, anseios, desafios e angústias do viver. Coloca aos gestores uma pergunta essencial: como profissionais/serviços podem colaborar na produção de (novos) horizontes de vida e de habilidades para lidar com problemas corriqueiros nessa fase? Para tal, é preciso ter em conta a temporalidade do cuidado, entendida como a articulação entre a experiência pretérita e a situação atual, que estruturam ideias, juízos e modos de ser e estar no mundo, e o futuro, que direciona a ação impulsionada pelos desejos e possibilidades de realizá-los ^10^.

Aqui cabe aventar a possibilidade de transformar a APS num espaço de aprendizagem de ser/viver ^17^ ^,^ ^18^. A atuação dos serviços na produção do eu seria assentada na dialogicidade ^19^ e na interseccionalidade como referencial interpretativo ^8^, devendo incorporar a integralidade do cuidado em uma perspectiva interdisciplinar que inclua a responsabilização compartilhada da equipe multiprofissional na mediação das necessidades desse segmento populacional.

Esse é apenas um dos desafios postos. Duas das UBS investigadas tinham grupos de adolescentes, voltados a usuários/as com “problemas de comportamento na escola/família” e transtornos mentais. A abordagem seguida pelas/os psicólogas/os correspondia a um modelo normalizador de condutas. O quantitativo de participantes costumava ser bastante baixo. Já os atendimentos individuais tendiam a provocar o abandono devido à periodicidade espaçada (pouca disponibilidade de profissionais frente à elevada demanda).

Há de se afirmar as nuances do cuidado dos mais jovens: são requeridos arranjos e manejos contextualizados e em ato, considerando-se as particularidades e demandas que emergem no cotidiano ^20^. Eles/as trazem percepções distintas sobre a oferta de grupos, ao mesmo tempo em que valorizam e desejam espaços de diálogo com pares mediados por profissionais para aprender, se informar, esclarecer dúvidas; afirmavam não se sentirem confortáveis em falar de si mesmos, de expor questões íntimas diante de outros jovens que não conhecessem, ou que conhecessem, mas não tivessem proximidade.

Outro ponto relevante é a escassez de iniciativas de educação em saúde em escolas do território adscrito. Alguns/mas entrevistados/as consideraram a presença de profissionais da UBS na escola estratégica para a divulgação dos serviços e a aproximação de potenciais usuários/as. Todavia, salienta Liz, 17 anos, quando ocorrem visitas às escolas, os temas e a abordagem adotados mostram-se distanciados dos interesses e questões juvenis. Para ela, o adultocentrismo obstaculiza a participação do jovem na promoção da saúde: “...*a palestra não é de um jeito que prende a gente e faz a gente querer participar. E aí, quando a gente quer participar, às vezes cortam a gente.* (...) *a maioria dos adultos não conseguem entender muito bem os mais jovens*”.

É nesse sentido que alguns entrevistados propuseram a criação de um serviço que produza saúde como bem-estar, por meio da sociabilidade juvenil, de expressões culturais e de práticas esportivas: “...*um espaço para conviver depois da escola, ficar com colegas da mesma idade, conhecer gente fora da escola e aprender outras coisas que a gente não consegue aprender na escola. Com atividades que a gente gosta, como jogos, assistir filme, dançar junto*...” (Júlia, 19 anos).

Em linha com experiências de incorporação de centros de juventude como estratégia descentralizada de promoção da saúde. Integrados no tecido social e subjetivo juvenil, são espaços simbolicamente atrativos que logram intervenções fluidas (oficinas, rodas de conversa, mentoria), em que o acolhimento, aprendizados para o bem-viver e a sociabilidade ocorrem de forma integrada ^21^ ^,^ ^22^.

### Problemas estruturais como barreira de acesso

“*Demora*” e “*fila de espera*” para a ocorrência de consultas, exames e encaminhamentos a outras especialidades foram consensualmente assinaladas como fatores de afastamento dos serviços públicos de saúde. Somam-se o absenteísmo e o déficit de “profissionais/médicos” como expressões das dificuldades colocadas a um processo de trabalho precarizado: “*Eles são sobrecarregados* (...) *uma hora tá esgotado psicologicamente* (...)*. Ele* [o profissional] *tem que fazer ‘Ah, só toma tal remédio’, pra atender 40*” (Alessandra, 17 anos).

Todavia, é preciso destacar que o problema central não é a longa espera e a consulta rápida *per si*, mas o desfecho insatisfatório. O entendimento é que, no ponto culminante do processo, o atendimento precisa produzir alguma correspondência com o esperado, tendo em vista a situação enfrentada. O atendimento significativo deve ser resolutivo, respeitoso, informativo com explicações (referem ao diagnóstico e tratamento) inteligíveis e que fazem sentido para o/a usuário/a, promovendo aprendizados em saúde.

“*Quando uma pessoa tá tirando 4 horas da vida dela, tá escolhendo cuidar da questão de saúde.* (...) *dependendo de como foi atendido nesses 15 minutos* [da consulta]*, se isso vai ser proveitoso, se isso vai me dar informação o suficiente pra eu querer voltar*...” (Vladimir, 20 anos).

Vários/as jovens ainda assinalaram que os problemas estruturais do sistema de saúde prejudicam o acompanhamento efetivo e adequado de seu estado de saúde/doença. Eles/as destacaram o quão problemático ou aflitivo é não conseguir atendimento num momento de crise ou problema agudo, sob risco de agravamento. O hiato entre a manifestação de um problema de saúde e a consulta agendada, além da ausência de seguimento do atendimento em casos de urgência, pode acarretar a utilização de serviços privados de baixo custo. É o caso de Marina, 17 anos, nos episódios de *“crise muito forte* [de gastrite] *porque às vezes não dá tempo,* (...) *eu preciso tratar! Se eu for no SUS eles vão me dá remédio e é isso. Ás vezes eu preciso de um encaminhamento pra fazer um exame de endoscopia e aí vai demorar não sei quanto tempo. Até lá já mudou - já posso tá pior, já posso tá melhor*”.

A avaliação do atendimento prestado em serviços de urgência também foi decisiva para a desistência de um uso regular do serviço público por alguns/mas jovens: acessa-se “*só em casos tipo muito grave, que a gente sabe que não vai ter como resolver*” (Alessandra, 17 anos), com o tempo ou com a automedicação ^23^. Além da elevada espera em um ambiente lotado, pesam o modelo queixa-conduta e a ausência de um diagnóstico (resposta/conclusão) sobre o mal-estar súbito. Os relatos trazem uma crítica consistente ao foco exclusivo na medicação para a resolução imediata do agravo, prescindido de um encaminhamento para posterior investigação. Eles/as ressaltam a importância de saber o que se tem/teve. Evidenciam, portanto, o sentido de não orientar as ações em saúde estritamente por uma apreensão do tipo causa-efeito ^10^, mesmo em um serviço de urgência.

Apesar de apontarem ser um problema generalizável à população de usuários do Sistema Único de Saúde (SUS), há uma percepção de não reconhecimento do “jovem” como interlocutor socialmente válido. Quando atravessado por opressões/vulnerabilidades, esse sentimento de invalidação pode agudizar: algumas falas colocam a desatenção do/a profissional à dor ocasionada em procedimentos sensíveis, como expressão de “desrespeito” à sua classe, sexualidade ou raça. A força empregada no manuseio de seus corpos parece-lhes traduzir a abjeção imputada. Acompanhada da namorada em uma coleta de sangue, Jussara, 17 anos, associou o “*preconceito comigo*” com o “*foi com tudo com a agulha*”. Dizem essas/es jovens que uma abordagem diligente requer a observação e a indagação sobre temores e desconfortos.

O atendimento descortês, desde a recepção, foi destacado por alguns/mas entrevistados/as como fator que contribui com o acabrunhamento nos serviços de saúde ou mesmo a desistência por buscar cuidado e/ou orientação (Quadro 2). Daí eles/as continuamente asseverarem a necessidade global de “acolhimento” nos serviços: “*Você vai chegar lá na recepcionista, vai fazer uma pergunta, talvez ninguém esteja* (...) *tá num ótimo dia, né? Mas existe aí a empatia pelo próximo, até porque ninguém sabe o que a gente passa, então, uma olhada diferente, uma resposta ignorante, já deixa aquela pessoa mal*” (Yolanda, 17 anos).

A “vergonha” deve também merecer atenção nos distintos contextos de atendimento. Se a perspectiva do constrangimento já os/as inibe, a sua materialização conforma o sentimento de “desconforto”, tantas vezes relatado. Tal aspecto reforça a importância do investimento valorativo na relação a ser produzida entre usuários/as e trabalhadores/as.

Esse tema integra a discussão sobre a falta de preparo profissional como elemento catalisador de obstáculos ao acesso, ao reconhecimento de necessidades e à realização de ações propostas no/pelo serviço público ^24^. Não podemos deixar à margem as dificuldades decorrentes da crescente adoção de dispositivos de controle, de captura do trabalho vivo, de endurecimento da gestão, de empobrecimento dos encontros entre trabalhadoras/es e entre trabalhadoras/es e usuárias/os que acontecem nas práticas de saúde na APS. Todavia, é preciso demarcar os encontros e movimentos potentes que evidenciam compromissos com o cuidado contextualizado e singular ^25^.

Estudos futuros podem avançar em uma análise interseccional do acesso, focalizando entrelaçamentos de vulnerabilidades e práticas discriminatórias, e de desrespeito nos serviços de saúde. Outro aporte relevante seria a diversificação de contextos. Embora realizada em territórios periféricos, a pesquisa pode não refletir as dinâmicas de acesso em regiões com diferentes configurações da rede de saúde. Ainda que os serviços disponíveis não atendessem às demandas locais, os componentes fundamentais estavam presentes. Acresce os equipamentos de cultura, de assistência social e ONGs, que moderaram as carências de serviços públicos voltados à juventude.

## À guisa de conclusão: alguns elementos para o aprimoramento do atendimento a jovens

Nessa derradeira seção, articulamos as significações das experiências de acesso dos/as jovens com guias produzidos por agências das Nações Unidas (Organização Mundial da Saúde - OMS, Fundo das Nações Unidas para a Infância - UNICEF, Organização Pan-Americana da Saúde - OPAS) ^26^ ^,^ ^27^ ^,^ ^28^, que propõem diretrizes para a qualificação dos serviços voltados a esse público. Tomando a atenção primária amigável ao jovem, o cuidado centrado na pessoa e a responsividade do sistema de saúde como pilares conceituais interconectados, elaboramos um conjunto de recomendações para instrumentalizar gestores e profissionais da APS na direção da produção cotidiana de espaços de vinculação, promoção de autonomia e garantia de direitos (Quadro 3).

**Quadro 3:** Recomendações para qualificar a atenção a adolescentes e jovens.

ABORDAGEM DE CUIDADO CENTRADO NA PESSOA
Construir uma conexão empática, iniciando o atendimento com conversas informais sobre a vida (interesses, cotidiano, escola). A escuta ativa contribui para validar sentimentos e experiências, investigar determinantes sociais da saúde e compreender aspectos relevantes à elaboração de um projeto de cuidado integral Explicitar as regras (e limites) sobre sigilo e consentimento para estabelecer a confiança necessária ao cuidado. Negociar o envolvimento da família, sem alienar o adolescente, viabiliza o respeito à sua autonomia progressiva. Também importa garantir momentos a sós como padrão de atendimento Usar linguagem clara (adaptada à idade) e não julgadora Adaptar a prática de atenção às especificidades do sujeito, já que as necessidades em saúde vão se modificando conforme o momento no percurso biográfico adolescente/jovem Promover literacia em saúde para fomentar a autonomia, especialmente decisões informadas, reconhecendo o adolescente como agente na gestão de sua saúde e bem-estar Redefinir a atuação do profissional como facilitador de saúde, priorizando o vínculo como o principal determinante de responsividade e da própria eficácia terapêutica. O jovem deve se sentir convidado a atuar como parceiro fundamental em um projeto de cuidado longitudinal
CAPACITAÇÃO E EDUCAÇÃO PERMANENTE
Instituir capacitações regulares focadas em competências de comunicação, ética da confidencialidade e condição e práticas juvenis Capacitação para a sensibilidade cultural e o não julgamento. O profissional deve identificar e suspender/dirimir preconceitos e valores morais que possam invalidar práticas e subjetividades juvenis Treinamento da equipe de apoio (portaria, recepção) em atendimento amigável e respeito à diversidade
ESCOLA COMO TERRITÓRIO ESTRATÉGICO DE SAÚDE
Promover ação intersetorial sistemática com profissionais e equipes nas escolas da área adstrita, com oferta de serviços clínicos *in loco* ou referenciados (saúde mental, saúde sexual e reprodutiva), realização de plantões de dúvida e rodas de conversa Desenvolver a capacitação de docentes e de gestores para identificar sinais de problemas psicossociais e estabelecer fluxos de encaminhamento para a UBS
PARTICIPAÇÃO JUVENIL NA GOVERNANÇA DO SERVIÇO DE SAÚDE
Instituir conselho consultivo de jovens usuários que se reúna periodicamente com a gestão da unidade para avaliar o serviço, propor melhorias (de acordo com culturas e necessidades juvenis locais) e produzir materiais educativos Capacitar jovens da comunidade como educadores de pares, que atuam como ponte entre a UBS e o território, facilitando a entrada de outros jovens no serviço Realizar grupos operativos na unidade ou no território (escolas, ONGs) focados em produções de si, projeto de vida e redução de danos
ARTICULAÇÃO INTERSETORIAL
Estabelecer parcerias com escolas, ONGs, grupos de pesquisas universitários para capacitações relativas a questões de juventude Utilizar espaços comunitários (equipamentos de cultura, escolas, ONGs e projetos sociais) para alocar estratégias transversais de cuidado, por meio de oficinas, mentoria e rodas de conversa sobre questões de saúde elegidas pelos/as jovens Flexibilizar a exigência de agendamentos prévios, facilitando o atendimento de demandas espontâneas e o autoencaminhamento juvenil

OBS: organizações não-governamentais; UBS: unidade básica de saúde.

Nessas recomendações buscamos alinhavar muitas das falas sobre atendimentos que traziam como conclusão ou justificativa que os/as profissionais “*não conseguem*” os/as “*entender*”. As proposições apresentadas para qualificar esses atendimentos podem ser sintetizadas na integração dos/as jovens como sujeitos com participação corresponsável no processo de cuidado. Os/As profissionais devem atuar como facilitadores/as, que oferecem opções e discutem decorrências, fomentando a sua agência ^3^ ^,^ ^29^. É nesse sentido que eles/as conferem centralidade ao sentido formativo da atenção à saúde, que deve colaborar para a construção de vidas mais felizes.

Uma maior horizontalidade relacional é tecida e estruturada na “conversa”, que impulsiona a compreensão da alteridade (chamada de “empatia”), que consolida o respeito às suas noções de saúde e explicações sobre si e o campo de possibilidades do projeto terapêutico a ser traçado, de modo a promover caminhos mais resolutivos de prevenção de agravos e da promoção da saúde.

A “conversa” é defendida como tecnologia fundamental para o cuidado em saúde também por favorecer uma dimensão afetivo-emocional. Confabulações sobre sentimentos e desafios cotidianos/existenciais conduzem a reflexões sobre si, fornecendo recursos para a autoemancipação e a formação de uma consciência mais aberta e indagadora ^19^. Tomando essa perspectiva em sua dimensão educativa, podemos evocar a formação para a vida: “*a educação é, antes de mais nada, uma viagem interior*” ^17^ (p. 101). Trata-se de um enfoque que contempla um repertório de competências para conduzir a uma relação saudável consigo mesmo/a e com o mundo.

Sem desconsiderar o crescente processo de precarização do trabalho na APS, sublinhamos que as tecnologias relacionais têm sido discutidas e experimentadas nesse contexto de atendimentos ^3^ ^,^ ^25^. Em complementaridade, há entre alguns/mas jovens o reconhecimento da potencialidade do modelo de atenção da Estratégia Saúde da Família (ESF), ressaltando o papel de agentes comunitárias/os de saúde. Foi sugerido incluir em suas visitas domiciliares momentos de escuta e aconselhamento de demandas e necessidades de saúde, como modo de favorecer a oferta de ferramentas para o cuidado de si ^30^.

Ademais, expressões artístico-culturais, esportivas e de lazer podem ser mobilizadas para o engajamento e a construção de conhecimentos que ressignifiquem práticas e noções sobre temas de saúde. Uma forma alternativa de “estar juntos”, que integra ação, pensamento e sentimento ^1^, e pode ser assimilada por profissionais em práticas processuais de educação em saúde ^10^. A construção de cuidado integral nesses moldes demanda a recriação de sua vida cotidiana. Abordagens criativas e lúdicas contrastam com a repetição e o automatismo das rotinas escolares e familiares. Esse tipo de ambiente permite que os sujeitos empreendam um processo educativo construído entre a dimensão perceptual-vivencial e sua práxis ^19^ ^,^ ^31^.

As interfaces de cuidado aqui apresentadas transversalizam a formação humana ao mover-se na ampliação de horizontes com a constituição de um usuário ativo e altivo, apossado de percepções sobre conceitos elementares para um projeto de saúde. Isso porque gerar processos criativos e solidários de melhoria da vida ^10^ imbrica-se com a produção da identidade, do autoconhecimento e de novas formas de atuar em sociedade.

## Data Availability

Os dados de pesquisa estão disponíveis mediante solicitação ao autor de correspondência.
